# Effect of experimentally introduced interaural frequency mismatch on sentence recognition in bilateral cochlear-implant listeners

**DOI:** 10.1121/10.0017705

**Published:** 2023-04-03

**Authors:** Miranda Cleary, Kristina DeRoy Milvae, Nicole Nguyen, Joshua G. W. Bernstein, Matthew J. Goupell

**Affiliations:** 1 Department of Hearing and Speech Sciences, University of Maryland, College Park, Maryland 20742, USA; 2 National Military Audiology and Speech Pathology Center, Walter Reed National Military Medical Center, Bethesda, Maryland 20889, USA micleary@umd.edu, klmilvae@buffalo.edu, nknguyen@umd.edu, joshua.g.bernstein.civ@health.mil, goupell@umd.edu

## Abstract

Bilateral cochlear-implant users experience interaural frequency mismatch because of asymmetries in array insertion and frequency-to-electrode assignment. To explore the acute perceptual consequences of such mismatch, sentence recognition in quiet was measured in nine bilateral cochlear-implant listeners as frequency allocations in the poorer ear were shifted by ±1.5, ±3, and ±4.5 mm using experimental programs. Shifts in frequency allocation >3 mm reduced bilateral sentence scores below those for the better ear alone, suggesting that the poorer ear interfered with better-ear perception. This was not a result of fewer active channels; deactivating electrodes without frequency shifting had minimal effect.

## 1. Introduction

Bilateral cochlear implants (BI-CIs) offer advantages over unilateral cochlear implants (CIs) for sound localization and speech understanding in noise (Smulders *et al.*, 2016). While having two CIs is usually beneficial, under challenging conditions, such as multiple competing talkers, some BI-CI users display a form of contralateral speech interference in that they perceive speech more poorly with bilateral inputs than with a unilateral input (Goupell *et al.*, 2018a). Analogous effects involving contralateral speech interference may also affect 10%–20% of older bilateral hearing-aid users (Jerger and Silverman, 2018).

Contralateral speech interference could be related to interaural asymmetries in electrode location or device programming. Because of the cochlea's tightly coiled structure, CI electrode arrays are often inserted only approximately one full basal turn of the cochlea relative to its full 2.5–2.75 turns. The array then delivers information about input frequencies between ∼0.2 and 8 kHz to locations tuned to higher frequencies in acoustic hearing, resulting in tonotopic frequency-to-place mismatch (Landsberger *et al.*, 2015). Additionally, surgical or anatomical considerations sometimes result in different insertion depths, such that one-third of BI-CI users are estimated to have at least three electrode pairs with >3 mm (∼75°) of interaural frequency mismatch (Goupell *et al.*, 2022). Asymmetric clinical deactivation of electrodes, due to facial nerve stimulation, for example, can also introduce interaural frequency mismatch because the full range of input frequencies is typically reallocated to remaining active electrodes, irrespective of the allocation on the opposite side.

Interaural frequency mismatch reduces bilateral word recognition scores in normal-hearing individuals listening to vocoder CI simulations when compared with conditions with no mismatch (Yoon *et al.*, 2011; van Besouw *et al.*, 2013; Fitzgerald *et al.*, 2017; Goupell *et al.*, 2018b). Yoon *et al.* (2011) additionally found that a 3-mm interaural mismatch—simulating a typical insertion on one side and shallower insertion on the other—reduced bilateral scores in quiet to below unilateral performance with the typical insertion simulation. Thus, the distorted speech in the more shifted ear interfered with perception, rather than providing supplemental information.

These vocoder studies are suggestive of a relationship between interaural frequency mismatch and contralateral speech interference, but they did not involve actual BI-CI listeners. Although it is not practical or ethical for researchers to physically relocate existing electrode arrays to manipulate interaural mismatch, it is possible to change interaural frequency alignment through adjustments to clinical programs. The present study, therefore, assessed whether bilateral sentence scores were influenced by manipulating frequency allocations in the poorer ear of BI-CI listeners, with scores for the unshifted better ear alone serving as a baseline. If a shifted signal in the poorer ear could be ignored, we hypothesized that bilateral scores would be no worse than for the better ear alone. This study tested sentence recognition in quiet (in preparation for future planned testing in noise), expecting that interference effects were unlikely to be seen under such favorable listening conditions. Surprisingly, interference effects were observed, increasing with degree of interaural mismatch.

## 2. Method

### 2.1 Participants

Nine post-lingually deafened adult BI-CI users participated (mean age: 67 years). Each participant was an experienced bilateral user of Cochlear-brand CIs, which are intended to have 22 active intracochlear electrodes, although in some cases, individual electrodes were clinically deactivated (Table 1). Participants used either the CP910, CP920, CP950, or CP1000 as their everyday CI sound processors. The poorer ear was determined from previously collected sentence recognition scores in quiet or by participant report (P01, P09) if right- and left-ear scores were within one percentage point. Computed-tomography-based electrode angular insertion depths available for six participants suggested minimal interaural mismatch (Table 1); there were few electrode pairs with mismatch >75°, the amount associated with functional consequences (Bernstein *et al.*, 2021). All participants passed a brief cognitive screening, scoring ≥26 on the Montreal Cognitive Assessment (MoCA) (Nasreddine *et al.*, 2005). Procedures were approved by the Institutional Review Board at the University of Maryland–College Park, and participants provided informed consent.

**Table 1.** Device and program details for individual participants.

Subject	Age (years)	Re-programed poorer ear	Internal CI device, array		Absolute angular insertion depth difference across electrode pairs, mean/min/max	Duration of CI use (years)		Total # of clinically activated electrodes		Deactivated electrodes		# of electrodes in baseline Δ0 program
			Left	Right		Left	Right	Left	Right	Left	Right	
P01	82	Left	CI24RE, Cont. Adv.^a^	CI24RE, Cont. Adv.	unavailable	12	6	20	20	1,2	1,2	10
P02	61	Left	CI24RE, Cont. Adv.	CI24RE, Cont. Adv.	22.7°/0°/61°	11	10	22	22	n/a^b^	n/a	11
P03	68	Left	CI512, Cont. Adv.	CI24RE, Cont. Adv.	29.4°/16°/81°	10	12	22	22	n/a	n/a	11
P04	73	Right	CI422, Slim Straight	CI24R(CS), Contour	16.7°/0°/40°	4	15	20	19	1,2	1,2,3	10
P05	68	Right	CI24RE, Cont. Adv.	CI24RE, Cont. Adv.	unavailable	13	16	22	19	n/a	8,16,21	11
P06	69	Left	CI512, Cont. Adv.	CI24RE, Cont. Adv.	21.3°/3°/87°	12	11	22	22	n/a	n/a	11
P07	64	Right	CI512, Cont. Adv.	CI24RE, Cont. Adv.	46.0°/27°/58°	11	16	21	21	1	1	11
P08	34	Left	CI24RE, Cont. Adv.	CI24RE, Cont. Adv.	unavailable	12	16	21	21	1	1	11
P09	82	Right	CI24R(CS), Contour	CI24RE, Cont. Adv.	23.4°/2°/65°	19	13	22	22	n/a	n/a	11

^a^ Contour Advance (Cont. Adv.).

^b^ Not applicable (n/a).

### 2.2 Procedure

#### 2.2.1 Stimuli

Institute of Electrical and Electronics Engineers (IEEE) Harvard sentences (IEEE, 1969) recorded by a male talker were used to measure sentence recognition. Each contained five different keywords.

#### 2.2.2 Experimental programs

**Fig. 1.**
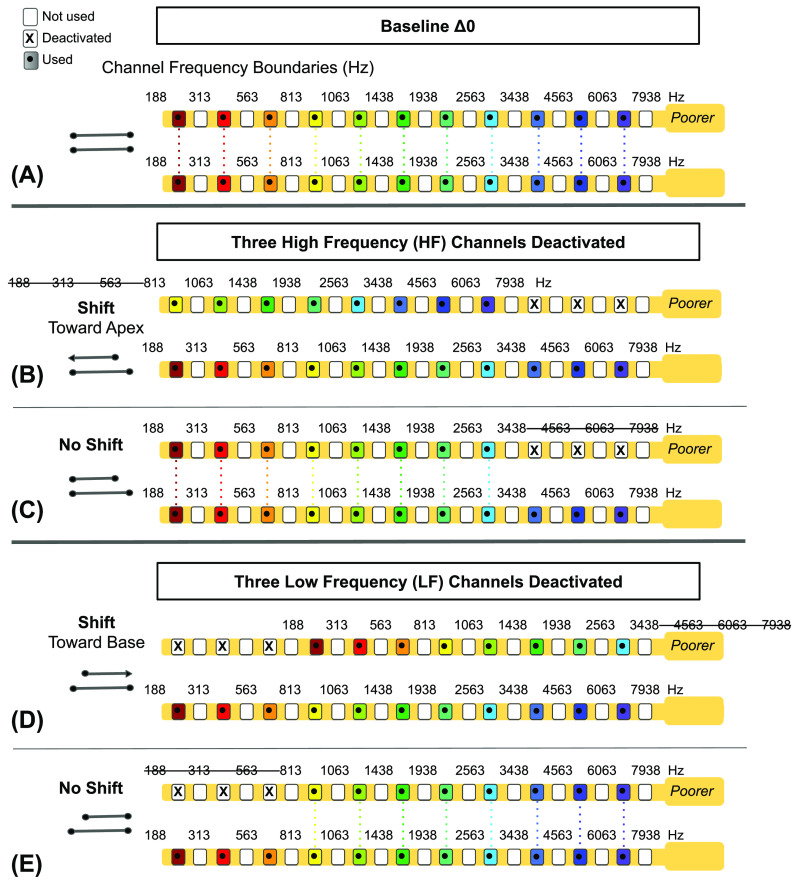
Example bilateral conditions. (A) Frequency-aligned programs with 11 active even-numbered channels were used in the baseline Δ0 condition. In the experimental conditions, one, two, or three (this example) electrodes were further deactivated in the poorer ear at the HF or LF end of the array. In each shift condition, frequency allocations were moved toward the apex (B), or base (D), by an equivalent number of electrodes, and unallocated frequencies at the end of the array were removed. In no-shift control conditions, (C) and (E), the same electrodes were deactivated but with no frequency shift.

A spectral-peak-picking CI sound processing strategy that activates subsets of electrodes per stimulation cycle can introduce differences in the signals across the ears at a given point in time (Gray *et al.*, 2021). To avoid this potential confound, clinical CI programming software (Custom Sound 5.2 or 6.2, Cochlear Ltd., Sydney, Australia) was used to convert each participant's clinical spectral-peak-picking programs to experimental programs that adopted a continuous interleaved sampling strategy (Wilson *et al.*, 1991) and frequency-aligned settings. These programs used every other electrode and set the number of maxima equal to the number of active electrodes, either 11 or 10 electrodes covering the clinical frequency range of 188–7938 Hz [Fig. 1(A)] or 188–6063 Hz (P01 only).

For any participant with clinically deactivated electrodes (Table 1), the corresponding electrodes in the opposite array were also deactivated. These programs with matched electrodes and frequency allocations served as the baseline “Δ0” condition. Only these baseline settings were used in the better ear.

The poorer-ear experimental programs were created by further deactivating one, two, or three electrodes at the low-frequency (LF) or high-frequency (HF) end of the array. These usually corresponded at the HF end to electrode 2 (1HF), electrodes 2 and 4 (2HF), or electrodes 2, 4, and 6 (3HF). At the LF end, these usually corresponded to electrode 22 (1LF), electrodes 22 and 20 (2LF), or electrodes 22, 20, and 18 (3LF).

The primary manipulation of interest was frequency shift. In the shift conditions [Figs. 1(B) and 1(D)], frequencies previously assigned in the baseline condition to now-deactivated electrodes were shifted to the nearest active electrodes. These electrodes were 2, 4, or 6 electrodes higher or lower, corresponding to shifts of approximately 1.5, 3, or 4.5 mm along the cochlea, using 0.75 mm as an approximate inter-electrode distance. Frequency allocations for all other electrodes were also shifted by the same number of electrodes, and displaced frequency bands were eliminated at the opposite end (i.e., no frequency compression was applied). For BI-CI listeners, the majority of whom have relatively well-matched arrays (Goupell *et al.*, 2022), shifted allocations mimic interaural frequency mismatch. Additional no-shift conditions [Figs. 1(C) and 1(E)] controlled for the loss of frequency information separate from the effects of shift, with frequencies previously assigned to deactivated electrodes simply removed from the signal. Only the 3LF and 3HF no-shift conditions were included because pilot testing showed negligible effects with fewer deactivated channels. A total of nine different programs (one baseline, six shift, two no-shift) were, therefore, created for the poorer ear.

The better ear was tested alone (one unilateral condition) or paired with each poorer-ear experimental program (nine bilateral conditions). Additionally, the poorer ear was tested alone with each experimental program (nine unilateral conditions), for a total of 19 conditions. The rationale for reprogramming only the poorer ear was to avoid making changes to the better ear, upon which BI-CI users often rely more heavily.

On the first day of testing, a research audiologist performed basic device diagnostics, such as checking device impedances and voltage compliances. They then fit the participant with the baseline experimental program first in the better ear and then in the poorer ear. Threshold and comfort levels were mostly left unchanged from those in the participant's clinical programs. For participant P08, one electrode's comfort “C” level was reduced to not exceed the compliance limit. Experimental programs were stored on laboratory clinical sound processors (N6, Cochlear Ltd.) dedicated to research purposes. Preprocessing features intended to improve speech perception in sound-field listening conditions (noise-reduction, audibility boosting, and SCAN options) were disabled. The default “standard” microphone mode, which is mostly omnidirectional, was used. Volume and sensitivity were left at each participant's preferred levels, usually the manufacturer defaults of 6 and 12, respectively.

#### 2.2.3 Stimulus presentation

Stimuli were presented via circumaural headphones (Sennheiser HD650, Hanover, Germany) placed around and over behind-the-ear CI sound processors (Goupell *et al.*, 2018a). During unilateral testing, the processor on the opposite side was removed or muted.

Testing occurred in a sound attenuated booth (International Acoustics Co., Bronx, NY). The stimulus level began at 65 dB-A, and the volume was then adjusted to a comfortably loud and interaurally balanced listening level. Adjustments were made primarily to the volume in the poorer ear (better-ear exceptions: P01, upward; P08 and P05, downward), and changes were limited to ±5 dB for a maximum of 70 dB-A. Level adjustment was done exclusively at the beginning of the session using the baseline programs and a recorded sentence with the same average root mean square amplitude as the test stimuli. Tasks were run using custom software (matlab, Natick, MA) running on a personal computer.

#### 2.2.4 Measurement of sentence recognition

On each trial, one sentence was presented to either a single CI or both CIs. The sentence was selected at random from the 720 IEEE sentences. The selection was made without replacement until the available sentences were exhausted for a given participant, at which point the selection began anew from the full set. Participants repeated the sentence aloud, while an experimenter scored which of the five keywords were correctly repeated. No feedback was provided.

For each of the 19 conditions, 60 sentences were tested in sets of 20 trials, one set per main testing block. For each of the three main testing blocks, a different randomization of the nine poorer-ear experimental programs was generated for each participant. The bilateral and unilateral conditions were counterbalanced as fully as possible across the three main testing blocks but were tested in groups of four experimental programs (one per sound-processor slot) to minimize the number of times the programs were changed and the CI processors and headphones were repositioned. Participants completed two main testing blocks on the first day of testing. On the second day, participants completed the third main testing block (except P08, who only completed two blocks) and then took a break to converse and socialize with the study team while wearing the 3HF shift program in the poorer ear and the baseline program in the better ear. (The 3HF shift condition in pilot testing appeared to be the most difficult.) After this additional experience, 60 more sentences were tested using the 3HF shift program. We also collected, either after all experimental testing or on a different day, sentence scores in quiet using the participant's own processors and settings. Thus, the testing required a total of at least 1440 sentences per participant. Although sentences were repeated, this repetition was randomly distributed across conditions.

### 2.3 Analysis

Linear mixed effect models (LMEMs) assessed the within-subject effects of experimental programs and number of inputs. Each model included random by-participant-varying intercepts and used the Satterthwaite *df* approximation to calculate denominators for *t* and *F* statistics. An α level of 0.05 was used.

## 3. Results

**Fig. 2.**
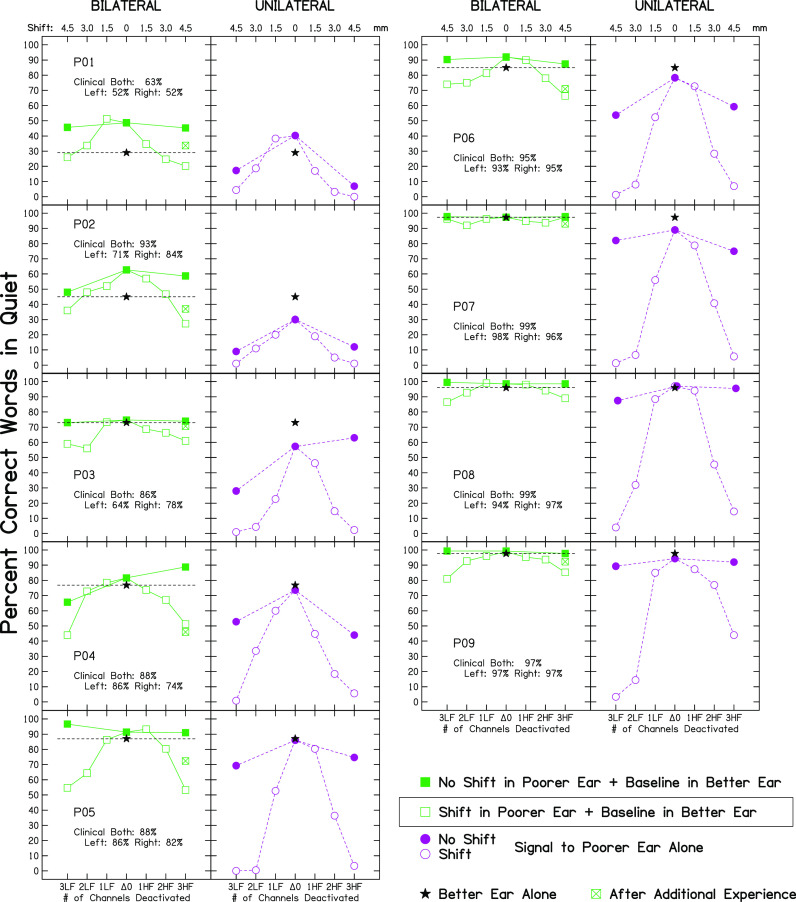
Sentence recognition scores for individual participants. Scores using their clinical programs are shown as text in individual panels. The box in the legend identifies the bilateral test condition of primary interest.

**Fig. 3.**
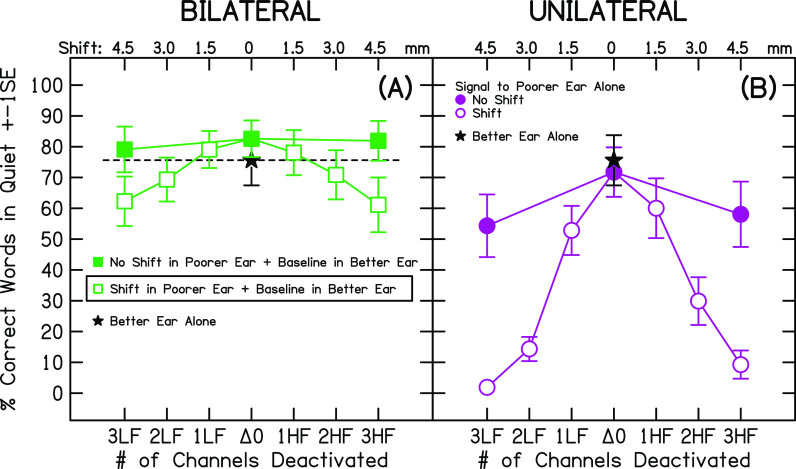
Average sentence recognition scores for bilateral [squares in (A)] and unilateral [poorer ear, circles in (B)] conditions. Filled symbols indicate control conditions with no frequency shift. Open symbols show shifted conditions. Unilateral scores for the better ear alone are shown by star symbols.

Sentence recognition scores are plotted as a function of the number of deactivated channels, shift condition, and ear of presentation in Fig. 2 (individuals) and Fig. 3 (group means). Most participants incurred only a small drop in bilateral performance for the baseline experimental programs (filled square at Δ0) compared to their everyday clinical programs (text labels in Fig. 2), despite the experimental program's use of novel every-other-electrode frequency allocations and more active channels. The average decrease of 7.1% (range: −3.7%–30.3%; everyday programs, *M* = 89.9%; experimental Δ0 programs, *M* = 82.8%) did not reach significance [*t*(9) = 2.13, *p* = 0.06].

The flat trajectory for the no-shift control conditions (filled squares at 3LF and 3HF) in Fig. 3(A) shows that eliminating three channels of information at either end of the frequency range in the poorer ear without shifting did not change bilateral performance [*F*(2,18) = 1.33, *p* = 0.29]. Frequency shifts in the poorer ear, however, had a clear detrimental effect when listening bilaterally (open squares). Scores decreased for eight of nine participants (all but P07; Fig. 2). The average decrease for a three-channel shift was approximately 20% [3LF = 20.9%, 3HF = 21.6%]. A LMEM analysis showed a main effect of experimental program [*F*(6,54) = 17.61, *p* < 0.001]. Nine pairwise comparisons—between the Δ0 reference level and each of the six experimental programs (3LF/2LF/1LF/1HF/2HF/3HF) and between shifts of the same magnitude but opposite direction (e.g., 3LF vs 3HF)—were conducted using a Bonferroni-corrected α = 0.0055. Two- and three-channel shifted conditions were significantly different from baseline (*p* < 0.0055), but one-channel shifts did not differ significantly from baseline. No significant differences were observed between shifts with the same magnitude but opposite direction. In summary, shifts of two and three channels (3 and 4.5 mm) in the poorer ear had a clear negative impact on bilateral scores, but shift direction did not influence this effect.

If the shifted signal in the poorer ear could be ignored, bilateral scores in the shifted conditions (open squares in Figs. 2 and 3) should have been no worse than unilateral better-ear alone scores (star symbols with dashed line in Figs. 2 and 3). This was not the case; bilateral scores in the two- and three-channel shifted conditions were lower, on average, than unilateral better-ear scores. A LMEM with experimental program treated as a categorical variable (six shifted programs in the bilateral condition plus the unilateral better-ear condition as the reference) showed a main effect of experimental program [*F*(6,54) = 13.66, *p* < 0.001]. Six pairwise comparisons between the unilateral better-ear score and bilateral scores for each experimental program (3LF/2LF/1LF/1HF/2HF/3HF) were conducted using a Bonferroni-corrected α = 0.008. Scores in both three-channel shifted conditions (3LF and 3HF) were significantly lower than unilateral better-ear scores (*p* < 0.008) by about 15% (3LF: 14.4%, 3HF: 15.1%), but the differences for one- and two-channel shifts did not reach significance. Thus, at least for three-channel (4.5-mm) shifts in the poorer ear, bilateral scores were poorer than unilateral better-ear scores, suggesting across-ear interference and an inability to ignore the shifted signal.

Although bilateral scores were the primary focus, the effects of frequency shift on unilateral scores were also of interest [open circles in Fig. 3(B)]. Previous vocoder research suggests that frequency shifts toward the base are more detrimental to unilateral sentence scores than equivalent shifts in millimeters toward the apex (e.g., Yoon *et al.*, 2011). Additionally, although vowel recognition by unilateral CI users is minimally affected by frequency shifts <3 mm in either direction (Fu and Shannon, 1999), the direction and degree of shift might differently impact perception of full sentences. A LMEM analysis of unilateral poorer-ear scores was conducted with shift direction (base/apex; categorical) and degree of shift (1/2/3; ordered discrete values) included as fixed effects together with their interaction. Although a main effect of shift was observed [*F*(1,45) = 108.6, *p* < 0.001], neither the main effect of direction (*p* = 0.35) nor the interaction (*p* = 0.98) was significant. Thus, shifts toward the base were not more detrimental than apical shifts. Additionally, pairwise comparisons showed that all levels of shift, including 1.5 mm, resulted in significantly lower unilateral scores than the baseline condition (corrected α = 0.017). For the unilateral control conditions, deactivating channels without shift significantly decreased scores relative to baseline [filled circles, Fig. 3(B)] [*F*(2,18) = 12.1, *p* < 0.001].

The x-marked squares in the bilateral panels of Fig. 2 show performance for the eight listeners retested in the bilateral 3HF shift condition following a 1-h acclimatization period. Performance improved significantly in this condition, reducing the interference magnitude [9.3% compared to 16.2% previously; *F*(1,8) = 6.03, *p* = 0.04]. The mean bilateral score (*M* = 64.5%), nevertheless, remained significantly lower than better-ear-alone pre-training scores (*M* = 73.8%) [*F*(1,8) = 6.99, *p* = 0.03].

## 4. Discussion

This study examined whether experimentally introduced interaural frequency mismatch influences sentence recognition in quiet for BI-CI participants. We found that shifts >3 mm reduced bilateral scores to levels poorer than for the better ear alone. Listeners were unable to attend only to the signal in their better ear when a distorted and frequency-shifted copy of the sentence was presented to their poorer ear.

Most previous BI-CI research exploring whether a poor input on one side can sometimes be disruptive to bilateral listening has examined the issue using simulations. Yoon *et al.* (2011), for example, simulated interaural frequency mismatch by presenting normal-hearing listeners with six-channel sine-vocoded sentences. When frequency allocations were matched between ears in a trained baseline condition, bilateral scores were better than for a single ear alone. In contrast, with a 3-mm frequency shift toward the apex in one ear, bilateral scores were no better than for the baseline unshifted single ear alone. Finally, with a 3-mm frequency shift toward the base, bilateral scores were significantly worse than for the baseline unshifted single ear alone. The present BI-CI data are largely consistent with such vocoder studies, although shifts toward the apex as well as toward the base were found to disrupt bilateral listening in the current study.

The ability to attend to one ear and ignore the other is influenced by the absolute and relative spectral-temporal characteristics of the two signals (Goupell *et al.*, 2021). Further research would be needed to determine why listeners found it difficult to ignore the shifted signal in the present study. It is unknown whether this result is specific to the frequency shift used here or if other types of distortion might yield similar results. Listeners were not explicitly told to ignore or attend to a particular ear. Instead, it was assumed that listeners would adopt the strategy that would most benefit task performance.

Frequency shift has been reported to hinder bilateral signal integration even when using both signals is the optimal strategy. Using six-channel vocoded speech and alternate channels presented to right and left ears, Siciliano *et al.* (2010) showed that after ten hours of training, normal-hearing listeners still could not integrate a 6-mm shifted signal in one ear with an unshifted complementary signal in the other ear to improve sentence scores compared to performance for the unshifted ear alone. Although there was important, albeit highly distorted, speech information in the shifted ear, it was not used to improve speech recognition.

Integrating information across two CIs offering dissimilar levels of benefit appears difficult and may play a role in how well binaural cues can be used to separate target speech from non-target input (Goupell *et al.*, 2018a). The 4.5-mm shifts in this study are outside the range of ±3 mm associated with monopolar electrical stimulation, beyond which significant decreases in BI-CI binaural processing occur (Goupell *et al.*, 2022), and, therefore, should be large enough to hinder signal integration. Recent vocoder research suggests that with background noise or competing talkers, even small interaural frequency mismatches of 1–2 mm might reduce BI-CI performance (Xu *et al.*, 2020).

The locus of the interference from interaural frequency mismatch is likely in the central auditory pathway. The reduced effect size for the 3HF/4.5-mm shift condition after further exposure (Fig. 2) suggests that some listeners can learn to reduce this interference, perhaps by focusing attention on only the better ear. The fact that some listeners improved more than others after exposure is of interest for further study. It should be noted that no control group was included to rule out possible effects of repeated testing. Additional testing with novel sentences or talkers would be useful to determine the type of learning taking place.

These results may contribute to understanding why less-than-expected benefit from two CIs is sometimes observed. If large interaural frequency mismatch can impact speech perception even in quiet, appropriate clinical approaches to real-world cases might include programming adjustments guided by such measures as computed-tomography estimates of electrode position (Bernstein *et al.*, 2021) to minimize such mismatch.
